# Exposure to Zinc Oxide Nanoparticles Disrupts Endothelial Tight and Adherens Junctions and Induces Pulmonary Inflammatory Cell Infiltration

**DOI:** 10.3390/ijms21103437

**Published:** 2020-05-13

**Authors:** Chen-Mei Chen, Meng-Ling Wu, Yen-Chun Ho, Pei-Yu Gung, Ming-Hsien Tsai, Alexander N. Orekhov, Igor A. Sobenin, Pinpin Lin, Shaw-Fang Yet

**Affiliations:** 1 Institute of Cellular and System Medicine, National Health Research Institutes, Zhunan 35053, Taiwan; chenmeichen@nhri.edu.tw (C.-M.C.); smokedplum@gmail.com (M.-L.W.); hoyenchun@gmail.com (Y.-C.H.); pygung@nhri.org.tw (P.-Y.G.); 2 National Institute of Environmental Health Sciences, National Health Research Institutes, Zhunan 35053, Taiwan; mhtsai@nhri.edu.tw; 3 Institute of Human Morphology, 3 Tsyurupa Street, Moscow 117418, Russia; a.h.opexob@gmail.com; 4 Laboratory of Medical Genetics, National Medical Research Center of Cardiology, 15A 3-rd Cherepkovskaya Street, Moscow 121552, Russia; igor.sobenin@gmail.com; 5 Graduate Institute of Biomedical Sciences, China Medical University, Taichung 40402, Taiwan

**Keywords:** zinc oxide nanoparticles, pulmonary infiltration, endothelial barrier, tight junctions, adherens junctions

## Abstract

Zinc oxide nanoparticles (ZnONPs) are frequently encountered nanomaterials in our daily lives. Despite the benefits of ZnONPs in a variety of applications, many studies have shown potential health hazards of exposure to ZnONPs. We have shown that oropharyngeal aspiration of ZnONPs in mice increases lung inflammation. However, the detailed mechanisms underlying pulmonary inflammatory cell infiltration remain to be elucidated. Endothelium functions as a barrier between the blood stream and the blood vessel wall. Endothelial barrier dysfunction may increase infiltration of immune cells into the vessel wall and underlying tissues. This current study examined the effects of ZnONPs exposure on endothelial barriers. ZnONPs exposure increased leukocyte infiltration in the mouse lungs. In endothelial cells, ZnONPs reduced the continuity of tight junction proteins claudin-5 and zonula occludens-1 (ZO-1) at the cell junctions. ZnONPs induced adherens junction protein VE-cadherin internalization from membrane to cytosol and dissociation with β-catenin, leading to reduced and diffused staining of VE-cadherin and β-catenin at cell junctions. Our results demonstrated that ZnONPs disrupted both tight and adherens junctions, compromising the integrity and stability of the junction network, leading to inflammatory cell infiltration. Thus, ZnONPs exposure in many different settings should be carefully evaluated for vascular effects and subsequent health impacts.

## 1. Introduction

With the advent of nanotechnology, nanomaterials have been used in a variety of applications. Of the nanomaterials, zinc oxide nanoparticles (ZnONPs) are frequently encountered in our daily lives, because of their common usage in many commercial and biomedical products [1]. However, extensive use of products containing ZnONPs will inevitably result in release of ZnONPs into the environment during production, transport, use and disposal processes [2]. Despite the benefits of ZnONPs in a variety of applications, many studies have shown potential health hazards of exposure to ZnONPs, including airway inflammation, spleen toxicity, neurotoxicity, etc. [2,3,4,5]. We reported previously, in mice, that a single high dose oropharyngeal aspiration for 2 days, or a repeated low dose aspiration of ZnONPs for 4 weeks, increases the total cell numbers in the bronchoalveolar lavage fluid (BALF) and pulmonary inflammation [6]. 

Vascular endothelium serves as a first line of defense against injury to blood vessels and the underlying tissues. Endothelium functions as a barrier and dynamically regulates the exchanges of substances between blood stream and the blood vessel wall; dysfunction of endothelial barrier may increase infiltration of blood proteins, toxic agents and immune cells into the vessel wall, resulting in various diseases [7]. The endothelial barrier function is achieved by a transcellular system and coordinated opening and closure of cell-cell junctions [8]. The endothelial paracellular junctions are primarily mediated by two junctional structures between the endothelial cells: tight and adherens junctions [8]. Tight junctions regulate paracellular movement of solutes and ions. Claudin family members are the major transmembrane constituents of tight junctions, while claudin-5 is the predominant claudin in endothelial cells [8], particularly at the alveolar-capillary barrier [9] and blood-brain barrier (BBB) [10]. Zonula occludens-1 (ZO-1) is the major scaffolding protein that stabilizes the tight junction barrier through coupling to the peri-junctional actin and myosin [8,11]. Adherens junctions play a key role in vascular integrity, and are important for vascular homeostasis [8]. Endothelial-specific vascular endothelial (VE)-cadherin (VE-cadherin) is the most important component of endothelial adherens junctions, and has a critical role in the intercellular interactions [12]. VE-cadherin-mediated endothelial cell contacts represent a barrier for extravasating leukocytes in vivo [13]. VE-cadherins associate as cis-dimers on the cell surface and form homophilic interactions between adjacent cells via extracellular domains [8,14]. VE-cadherin contains five extracellular domains (EC1-5), a transmembrane domain and a cytoplasmic domain. The EC1 domain has been shown to affect adhesion and clustering of VE-cadherins [14]. In addition, the cytoplasmic tail of VE-cadherin links to the cytoskeleton by binding directly and indirectly with various catenins, including β-catenin [8,15]. Targeting either the extracellular domain or the cytoplasmic tail deleteriously affects the junctional strength and leads to vascular permeability [16].

Two main pathways have been proposed underlying inflammatory cell infiltration due to endothelial dysfunction: transendothelial channels and para-endothelial cell junctions, a process also known as diapedesis [8,17]. Given that ZnONPs induce pulmonary inflammation and that inflammatory cell infiltration into the lungs is one of the critical events in causing lung disorders, it is important to further understand the barrier functions of the endothelium in the lungs and the underlying mechanisms. A previous study showed that ZnONPs increase endothelial permeability; however, the increases of permeability are inversely correlated with cell viability [18]. As such, it is not clear whether ZnONPs-increased permeability is due to impaired cell survival or other mechanisms.

In this study, we examined the effects of ZnNOPs exposure on vascular functions, particularly on endothelial barriers, using in vitro and in vivo models. We showed that ZnNOPs exposure in mice increased inflammatory cell infiltration in the lungs. Furthermore, ZnONPs exposure impaired endothelial paracellular barriers, thereby compromising the integrity and stability of the junction network, leading to inflammatory cell infiltration. Thus, ZnONPs exposure in many different settings should be carefully evaluated for vascular effects and subsequent health impacts. 

## 2. Results

### 2.1. Exposure of Mice to ZnONPs Induces Pulmonary Inflammatory Cell Infiltration

**Figure 1:**
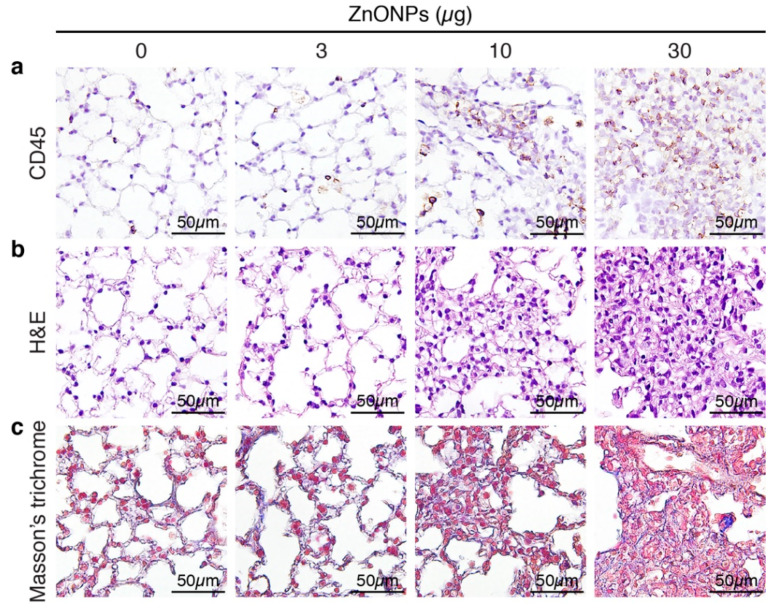
Repeated exposure of mice to zinc oxide nanoparticles (ZnONPs) induces inflammatory cell infiltration and fibrosis in the lungs. Mice were subjected to repeated exposure to different doses of ZnONPs twice a week for 4 weeks. Lung sections were stained with (**a**) leukocyte marker CD45 (brown) for inflammatory cell infiltration, (**b**) H&E staining for morphology, and (**c**) Masson’s trichrome (blue) for collagen.

**Figure 2:**
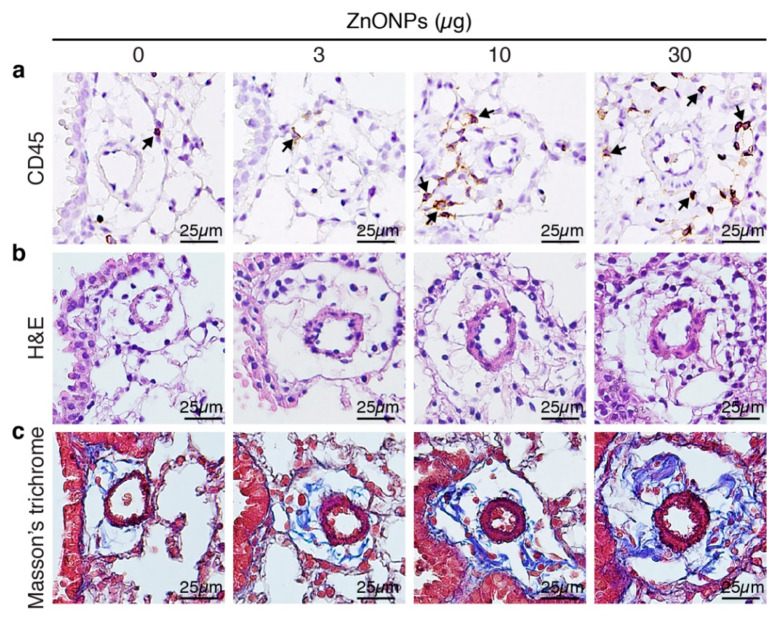
Repeated exposure of mice to ZnONPs enhances perivascular leukocyte infiltration, wall thickening and collagen deposition of the arterioles in the lungs. Mice were subjected to repeated exposure to different doses of ZnONPs twice a week for 4 weeks. Lung sections with arterioles were stained with (**a**) leukocyte marker CD45 (brown) for inflammatory cell infiltration (arrows), (**b**) H&E for morphology, and (**c**) Masson’s trichrome (blue) for collagen.

Aspiration of ZnONPs in mice induces pulmonary inflammation with an increased total cell number in BALF [6]. Further histological analysis revealed that repeated exposure of ZnONPs (0, 3, 10 or 30 µg/mouse, twice a week) for 4 weeks, dose-dependently increased inflammatory cell infiltration in the lungs (Figure 1a and Supplementary Figure S1a), accompanied with alveolar septa thickening (Figure 1b and Supplementary Figure S1b) and interstitial and bronchocentric patterns of fibrosis (Figure 1c). In addition, ZnONPs enhanced leukocyte infiltration in the perivascular space of arterioles (Figure 2a and Supplementary Figure S1c) with arteriolar wall thickening (Figure 2b and Supplementary Figure S1d) and increased collagen deposition (Figure 2c).

### 2.2. ZnONPs Increases Endothelial ICAM-1 Expression and Permeability

**Figure 3:**
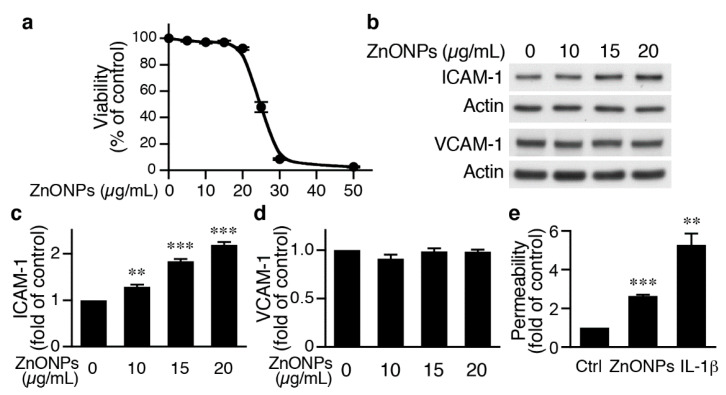
ZnONPs increase intercellular adhesion molecule-1 (ICAM-1) expression and permeability in human umbilical vein endothelial cells (HUVECs). (**a**) HUVECs were treated with increasing concentrations of ZnONPs (0, 5, 10, 15, 20, 25, 30 and 50 µg/mL) and cell viability measured after 24 h (*n* = 4, each). (**b**) HUVECs were treated with different concentrations of ZnONPs (0, 10, 15 and 20 µg/mL) for 24 h and total proteins prepared for Western blot analysis to detect ICAM-1 and vascular cell adhesion molecule-1 (VCAM-1) expressions. Actin was used as a loading control. (**c**) Quantification of ICAM-1 (*n* = 4 each group, ** *p* < 0.01, *** *p* < 0.001 vs. control). (**d**) Quantification of VCAM-1 (*n* = 4 each group). (**e**) HUVECs were treated with vehicle (control), ZnONPs (20 μg/mL) or IL-1β (10 ng/mL) for 24 h and permeability measured using FITC-Dextran (*n* = 3 each group, ** *p* < 0.01, *** *p* < 0.001 vs. control).

To begin to investigate the underlying mechanisms of ZnONPs-mediated leukocyte infiltration, we determined the effects of ZnONPs on endothelial cell function. We first treated human umbilical vein endothelial cells (HUVECs) with different concentrations of ZnONPs, and determined cell viability after 24 h. Treatment of HUVECs with ZnONPs up to 20 µg/mL caused no or minimal effect on viability (Figure 3a). However, a small increase to 25 µg/mL significantly decreased cell viability to 41.3 % (*p* < 0.01 vs. control) and higher concentrations (30 or 50 μg/mL) severely reduced viability to 7.5% and 2.4%, respectively (Figure 3a). As ZnONPs have been shown to elicit endothelial inflammatory responses, we examined expressions of adhesion molecules intercellular adhesion molecule-1 (ICAM-1) and vascular cell adhesion molecule-1 (VCAM-1), two inflammatory indicators of endothelial cells [19]. ZnONPs at 10, 15 or 20 μg/mL significantly increased ICAM-1 expression (Figure 3b,c). Intriguingly, ZnONPs did not affect VCAM-1 expression, even up to 20 μg/mL (Figure 3b,d).

We next examined whether ZnONPs affected endothelial permeability. Permeability assays showed that compared with control, ZnONPs at 20 μg/mL, a concentration without affecting cell survival, significantly increased permeability by 2.6-fold, while the inflammatory mediator IL-1β served as a positive control (Figure 1e). These data suggest that ZnONPs impaired endothelial barrier functions.

### 2.3. ZnONPs Disrupt Endothelial Tight Junctions

**Figure 4:**
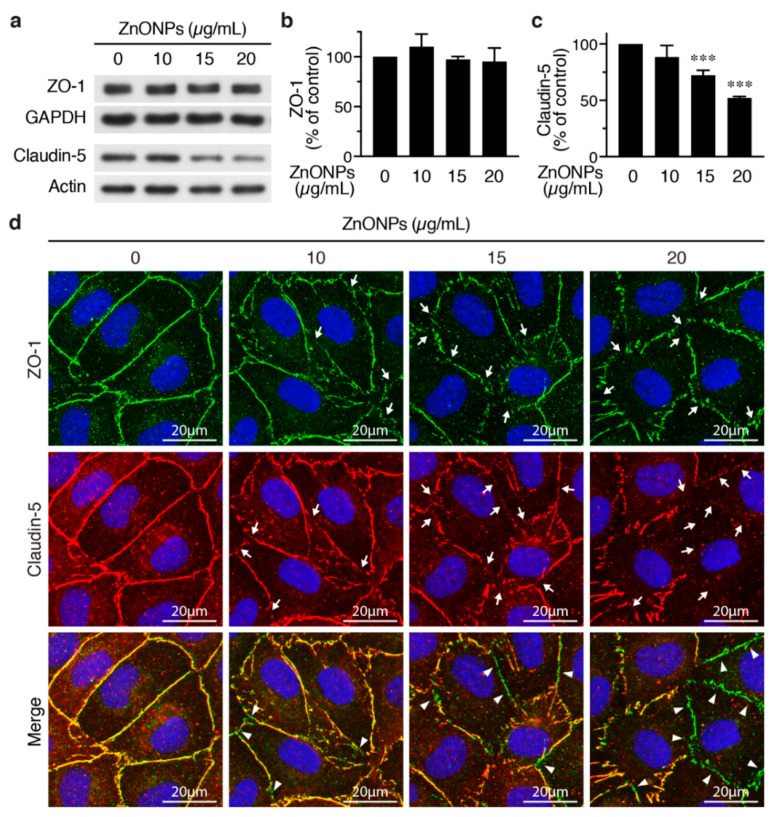
ZnONPs disrupt endothelial tight junctions. (**a**) HUVECs were treated with different concentrations of ZnONPs (0, 10, 15 and 20 µg/mL) for 24 h and total proteins prepared for Western blot analysis to detect zonula occludens-1 (ZO-1) and claudin-5. GAPDH or actin were used as loading controls. (**b**) Quantification of ZO-1 (*n* = three per group, no significant difference vs. control). (**c**) Quantification of claudin-5 (*n* = five per group, *** *p* < 0.001 vs. control). (**d**) Immunofluorescence staining of HUVECs treated as in (a) to detect ZO-1 (green, top row) and claudin-5 (red, middle row). Cell nuclei were stained blue with DAPI. Merged images of ZO-1 and claudin-5 are shown in bottom row. Yellow color indicates co-staining of ZO-1 and claudin-5. Arrows indicate loss of staining of ZO-1 or claudin-5 at the cell-cell junctions, while arrowheads denote tight junction segments where only ZO-1 was still present (bottom row, green).

We next set out to evaluate the effects of ZnNOPs on endothelial paracellular junctions. Western analysis showed that, although 20 μg/mL of ZnNOPs increased HUVEC permeability, it did not alter the expression level of the tight junction component ZO-1 (Figure 4a,b). Immunofluorescence staining revealed continuous staining of ZO-1 along cell-cell junctions in the absence of ZnONPs (Figure 4d, top row, left panel). Interestingly, exposure to ZnONPs (10 μg/mL) caused discontinuity of ZO-1 staining at the junctions and the disruption became more severe at higher concentrations of ZnONPs (15 and 20 μg/mL) (Figure 4d, top row, arrows). These results indicate that ZnONPs disrupt the continuous distribution of ZO-1 at the junctions, despite not affecting the ZO-1 expression level.

In contrast to ZO-1, ZnONPs at 15 and 20 µg/mL significantly reduced endothelial-specific tight junction protein claudin-5 expression to 72.3% and 52.2%, respectively (Figure 4a,c). Immunostaining showed a continuous distribution of claudin-5 along cell-cell junctions in control cells (Figure 4d, middle row, left panel). Although 10 µg/mL ZnONPs only slightly reduced claudin-5 level by Western blotting (Figure 4a,c), we observed a discontinuous distribution of claudin-5 at the junctions (Figure 4d, middle row, arrows). Higher concentrations of ZnONPs further decreased junctional distribution of claudin-5 (Figure 4d, middle row, arrows). Merged images revealed an overlapping expression of ZO-1 and claudin 5 at cell junctions (yellow color) in the control cells (Figure 4d, bottom row, left panel). The number of green segments (ZO-1) at the junctions positively correlated with concentrations of ZnONPs (Figure 4d, bottom row, arrowheads), indicating presence of ZO-1 but not claudin-5, which might be due to reduced claudin-5 expression. These results suggest that ZnONPs alter junctional distribution of ZO-1 and decrease expression level of claudin-5, leading to disruption of endothelial tight junctions.

### 2.4. ZnONPs Disrupt Endothelial Adherens Junctions

**Figure 5:**
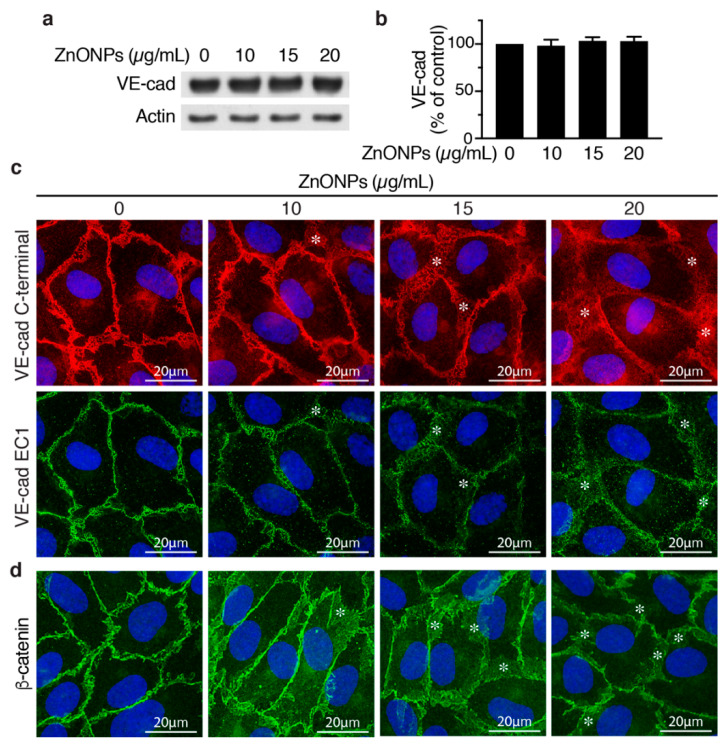
Effects of ZnONPs on adherens junctions in HUVECs. (**a**) HUVECs were treated with different concentrations of ZnONPs (0, 10, 15 and 20 µg/mL) for 24 h, and total proteins prepared for Western blot analysis to detect vascular endothelial (VE)-cadherin. Actin was used as loading control. A representative immunoblot is shown. (**b**) Quantification of VE-cadherin (*n* = 3, each). Data are mean ± SE, Student’s *t*-test shows no significant difference. (**c**) Representative images of double immunofluorescence staining of HUVECs with antibodies against VE-cadherin c-terminal (red) and EC1 domain (green). Cell nuclei were stained with DAPI (blue). (**d**) Distribution of β-catenin (green) after treatment with ZnONPs. *, diffused and disorganized expression of VE-cadherin or β-catenin at the cell-cell junctions.

To evaluate whether ZnONPs affect endothelial cell adherens junctions, we examined VE-cadherin expression by Western blotting and immunostaining in HUVECs. ZnONPs up to 20 μg/mL did not affect VE-cadherin expression level in HUVECs (Figure 5a,b). Immunostaining with VE-cadherin C-terminal antibody revealed that, in control cells, VE-cadherin distributed to the junctions in a tight and well-organized manner (Figure 5c, left column, top panel). In contrast, ZnONPs rendered VE-cadherin become loosely membrane-bound and diffused at the cell-cell junctions (Figure 5c, top row, *). Furthermore, ZnONPs, particularly at the higher dose of 20 μg/mL, increased cytosolic VE-cadherin distribution, as indicated by increased intensity of red fluorescence in the cytosol (Figure 5c). Correspondingly, immunostaining with antibody against VE-cadherin EC1 domain showed that ZnONPs reduced VE-cadherin distribution to cell-cell junctions and the continuity at the cell junctions (Figure 5c, bottom row). Together, these results suggest that ZnONPs treatment elicited VE-cadherin internalization, and resulted in the disrupted distribution of VE-cadherin at adherens junctions.

Given that the linkage of VE-cadherin’s cytoplasmic domain with actin cytoskeleton via catenins is crucial for stability of junctions, we examined junctional β-catenin to evaluate the effect of ZnONPs on adherens junction structures. ZnONPs treatment not only reduced but also rendered junctional β-catenin become diffused and away from the cell-cell junctions (Figure 5d), similar to that of VE-cadherin (Figure 5c). Collectively, immunostaining results indicate that ZnONPs destabilize endothelial adherens junctions by disrupting VE-cadherin and β-catenin localization at the cell-cell junctions.

### 2.5. ZnONPs Activate VEGFR2 and Render β-Catenin Dissociation from Membrane-bound VE-cadherin

**Figure 6:**
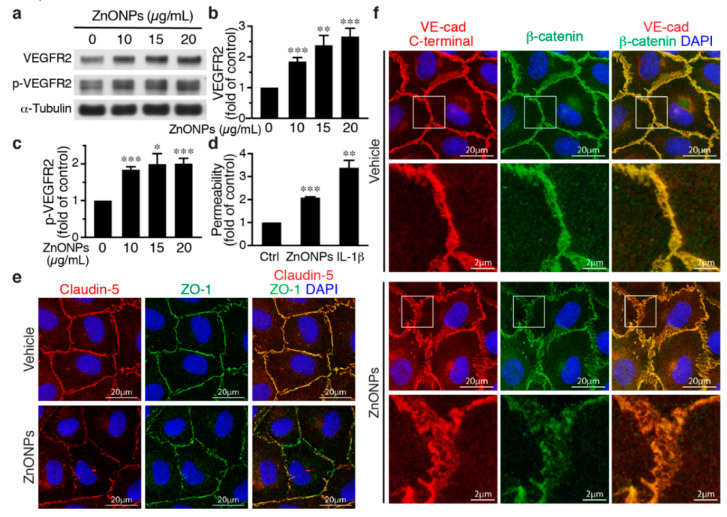
Effects of ZnONPs on vascular endothelial growth factor (VEGF) receptor 2 (VEGFR2), permeability, and VE-cadherin/β-catenin complex in endothelial cells. (**a**–**c**) HUVECs were grown in an endothelial cell growth medium-2 (EGM2) medium and then treated with ZnONPs for 24 h before analyses. (**a**) Western blotting was performed to detect total and phosphorylated VEGFR2. α-Tubulin was used as a loading control. A representative blot is shown. (**b**,**c**) Quantification of VEGFR2 and phospho-VEGFR2 (p-VEGFR2), respectively. (**d**) HUVECs were quiesced with starvation medium (without growth factors including VEGF). Permeability of quiesced HUVECs following 24 h of treatment with vehicle, ZnONPs (15 μg/mL) or IL-1β (10 ng/mL) was measured (*n* = 3). (**e**,**f**) HUVECs were quiesced with starvation medium and then treated with ZnONPs for 24 h before analyses. (**e**) Representative images of immunofluorescence double staining for claudin-5 (red), ZO-1 (green), and merged images (yellow) in quiesced HUVECs. (**f**) Representative images of immunofluorescence double staining with antibodies against VE-cadherin C-terminal (red) and β-catenin (green) of quiesced HUVECs treated with vehicle or ZnONP (15 μg/mL). Cell nuclei were stained with DAPI (blue). Merged images were used to evaluate the co-localization (yellow). Magnification of white boxes from the respective panels are shown in the lower row. Data are mean ± SE, Student’s t-test, * *p* < 0.05, ** *p* < 0.01, *** *p* < 0.001 vs. control.

Activation of the vascular endothelial growth factor (VEGF)-VEGF receptor 2 (VEGFR2) axis has been shown to facilitate VE-cadherin internalization [20,21]. We thus investigated the role of VEGFR2 in the effect of ZnONPs on endothelial junctions. ZnONPs increased not only total VEGFR2 but also VEGFR2 phosphorylation/activation (Figure 6a–c). Since the HUVEC growth medium contains VEGF, to rule out the possibility that this is due to VEGF in the medium, HUVECs were starved/quiesced in medium without VEGF before ZnONPs treatment. Under starvation conditions, ZnONPs maintained its ability to increase endothelial permeability (Figure 6d), suggesting that ZnONPs-mediated endothelial paracellular barrier alterations were likely independent of VEGF. Supporting this notion, ZnONPs not only elicited discontinuity of the tight junction proteins claudin-5 and ZO-1 (Figure 6e), but also caused diffused VE-cadherin staining at the cell-cell junctions (Figure 6f, left column).

Furthermore, dissociation of β-catenin from membrane-bound VE-cadherin following ZnONPs exposure was identified in starved HUVECs. Merged images of double immunostaining revealed that VE-cadherin and β-catenin were well colocalized at the cell-cell junctions without ZnONPs treatment (Figure 6f, vehicle). In the merged images from HUVECs treated with ZnONPs, the red-to-orange, rather than the strong yellow color, indicated that β-catenin might dissociate from VE-cadherin and contribute to ZnONPs-induced endothelial permeability (Figure 6f).

### 2.6. AhR-dependent and Independent Pathways Contribute to ZnONPs-mediated Endothelial Dysfunction

**Figure 7:**
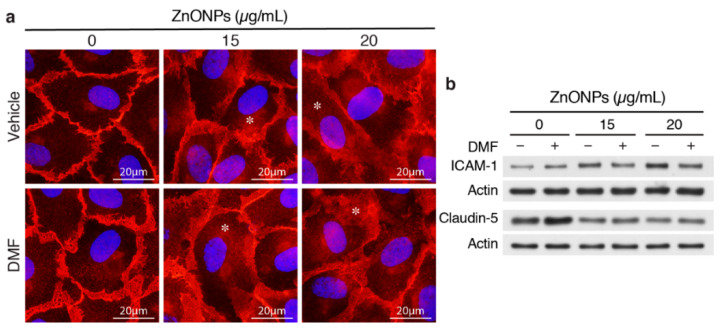
Aryl hydrocarbon receptor (AhR) inhibitor 3′,4′-dimethoxyflavone (DMF) abolishes ZnONPs-induced ICAM-1 but not claudin-5 expressions or alters VE-cadherin junctional distribution. HUVECs were pretreated with 10 µM DMF, before treatment with ZnONPs for 24 h. (**a**) Representative images of immunofluorescence staining with antibody against VE-cadherin c-terminal domain (red) are shown. Cell nuclei were stained with DAPI (blue). *, diffused cytosolic distribution of VE-cadherin. (**b**) Western blotting was performed to detect ICAM-1 and claudin-5 expressions. Actin was used as a loading control. A representative of at least four experiments is shown.

We reported previously that ZnONPs-induced pulmonary inflammation is mediated through inflammatory cytokine–indoleamine 2,3-dioxygenase (IDO1)–aryl hydrocarbon receptor (AhR) loop in the lung macrophages and epithelial cells [6]. We therefore assessed whether AhR plays a role in ZnONPs-mediated endothelial dysfunctions. Interestingly, AhR antagonist 3′,4′-dimethoxyflavone (DMF) did not inhibit ZnONPs-elicited diffused membrane and/or cytosolic distribution of VE-cadherin (Figure 7a). DMF suppressed ZnONPs-induced ICAM-1 expression, but did not rescue ZnONPs-decreased expression of claudin-5 (Figure 7b). These results suggest that ZnONPs induce ICAM-1 expression via AhR pathway whereas ZnONPs elicit alterations of endothelial junctions through mechanisms other than AhR pathway.

### 2.7. ZnONPs Exposure Impairs Vascular Integrity in the Mouse Pulmonary Arterioles and Alveoli

**Figure 8:**
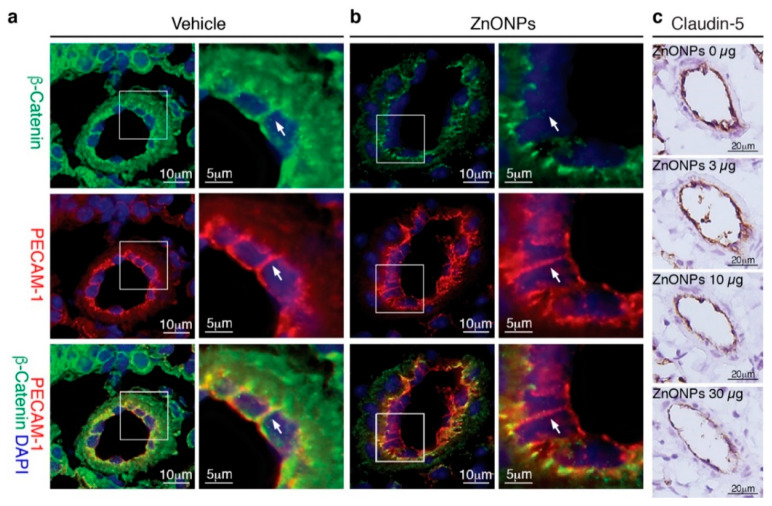
Repeated exposure to ZnONPs reduces β-catenin and claudin-5 in the pulmonary arteriolar endothelial junctions. Pulmonary arterioles from lung sections of mice repeatedly exposed to various doses of ZnONPs (0, 3, 10 and 30 µg) were subjected to histological analysis. Immunofluorescence double staining of platelet endothelial cell adhesion molecule 1 (PECAM-1) (red) and β-catenin (green) and DAPI (blue) for nuclei of pulmonary arterioles from mice exposed to (**a**) vehicle or (**b**) 30 µg ZnONPs. Merged images are shown in the bottom row and yellow color indicates overlapping expression. Magnified boxed areas are shown in the right column. White arrows indicate endothelial cell-cell boundaries. (**c**) Claudin-5 immunohistochemistry staining (brown) of pulmonary arterioles.

Our results showed that ZnONPs disrupt endothelial junction structures in vitro, we next examined whether exposure to ZnONPs affected vascular integrity in vivo. Platelet endothelial cell adhesion molecule 1 (PECAM-1) is expressed over the entire endothelial cell surface, including adherens junctions, and mediates both homotypic and heterotypic cell adhesion [8,22]. PECAM-1 is required for transendothelial migration of leukocytes [23]. To examine the expression and distribution of β-catenin at endothelial adherens junctions in the mouse pulmonary arterioles (Figure 8a, green, arrow), the PECAM-1 antibody is used to help identify endothelial cell-cell junctions (Figure 8a, red, arrow) in double immunostaining. Merged images of yellow color at the endothelial cell-cell junctions demonstrated colocalization of PECAM-1 and β-catenin in the control arterioles (Figure 8a, yellow, arrow). Repeated exposure to ZnONPs (30 µg) for 28 days did not affect PECAM-1 level at the endothelial cell-cell junctions in the lung arterioles (Figure 8b, red, arrow). Consistent with in vitro results, ZnONPs reduced the β-catenin presence at the cell-cell junctions to an almost undetectable level (Figure 8b, green, arrow). The red color at the cell-cell junctions in the merged images further demonstrated the disappearance of β-catenin (Figure 8b, arrow).

**Figure 9:**
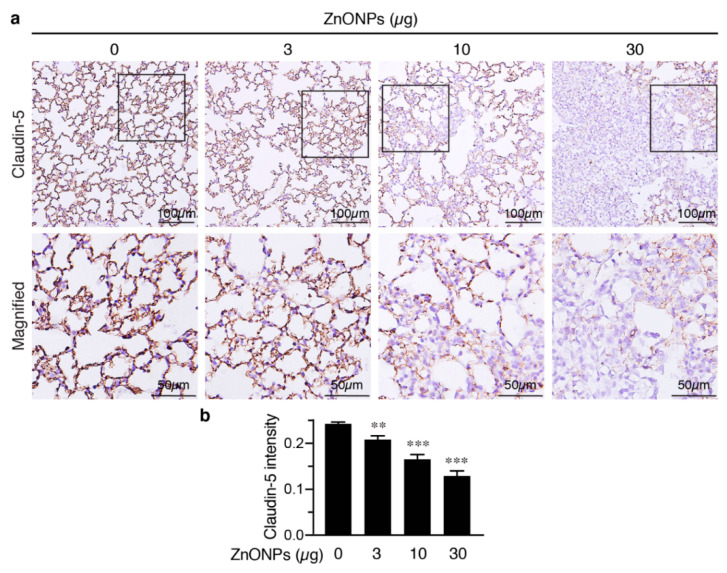
Repeated exposure to ZnONPs reduces claudin-5 in the alveolar endothelial junctions in the mouse lungs. (**a**) Top row, immunostaining of claudin-5 (brown) of lung sections from mice repeatedly exposed to various doses of ZnONPs. Bottom row, magnified images from boxed areas in (a). (**b**) Claudin-5 expression in DAB staining was quantified using optical density across tissue positive pixels (*n* = 8 each). Data are mean ± SE, Student’s t-test, ** *p* < 0.01, *** *p* < 0.001 vs. control.

We next evaluated the effects of ZnONPs on tight junction protein claudin-5 in the mouse lungs. Claudin-5 was highly expressed in the endothelial cell-cell junctions in the control pulmonary arterioles (Figure 8c, 0 µg) and exposure to ZnONPs reduced claudin-5 level in a dose-dependent manner (Figure 8c). As in arterioles, claudin-5 was highly expressed in the alveolar endothelial cell-cell junctions in the control lungs (Figure 9a, left column). Exposure to ZnONPs dose-dependently reduced claudin-5 expressions in alveolar septa (Figure 9a,b). At a low dose of 3 μg, ZnONPs elicited visible fragmented junctional distribution and a reduced level (by 14.3% vs. control) of claudin-5 (Figure 9a,b). Compared with control, ZnONPs at 10 and 30 µg severely reduced presence of claudin-5 by 31.9% and 46.9%, respectively (Figure 9a,b). Collectively, these results indicate that exposure to ZnONPs compromises the vascular integrity of the pulmonary arterioles and alveoli in mice.

## 3. Discussion

We demonstrated in this study that ZnONPs impair endothelial paracellular barriers by decreasing the expression level and/or distribution of tight and adherens junction proteins at the cell junctions, thereby reducing the integrity and stability of the junction network, leading to barrier dysfunction, which might in turn facilitate inflammatory cell infiltration via paracellular junctional opening. ZnONPs disrupted endothelial tight junctions by affecting at least two tight junction proteins, fragmented membrane-association of ZO-1, and reduced level and discontinuous distribution of claudin-5 at the junctions. At the adherens junctions, ZnONPs elicited VE-cadherin internalization from membrane to cytosol and dissociation with β-catenin, which links adherens junctions to cytoskeleton via binding to VE-cadherin C-terminal domain. In the mouse lungs, ZnONPs-induced disruption of endothelial junctions correlated with the level of leukocytes extravasation, severity of alveolar septal thickening, and collagen deposition in the lungs.

In an in vitro BBB model, silver nanoparticles and titanium dioxide nanoparticles disrupt tight junction proteins claudin-5 and ZO-1 and increase endothelial permeability [24]. Our results are similar in that ZnONPs reduced continuity of claudin-5 and ZO-1 at the tight junctions. ZnONPs also significantly decreased claudin-5 expression level (Figure 4). Interestingly, a recent study showed that small silica nanoparticles modulate the epithelial paracellular barrier via impairing the stability of tight junction network [25]. Together, it is tempting to speculate that tight junctions might be a common target of nanoparticles. Despite that ZnONPs did not affect expression level of the major adherens junction protein VE-cadherin, ZnONPs elicited VE-cadherin internalization from membranes/junctions to cytosol. This finding was supported by immunostaining with an antibody against either C-terminal or extracellular EC1 domain (Figure 5). Further supporting disorganization of adherens junctions induced by ZnONPs, a similar effect was observed on the junctional scaffolding protein β-catenin (Figure 5). β-Catenin binding to VE-cadherin is essential for the strength of cadherin-based adhesion, and its dissociation destabilizes adherens junctions [26,27]. Our findings that ZnONPs caused dissociation of VE-cadherin and β-catenin from cell-cell junctions are in line with the report that titanium dioxide nanoparticles bind and disrupt VE-cadherin homophilic interaction, resulting in loss of interaction between VE–cadherin and β-catenin, leading to endothelial cell leakiness [28]. Nevertheless, alterations of endothelial tight and adherens junctions may not occur simultaneously. Decreased VE-cadherin (cadherin-5) has been reported in a human diabetic retina, with normal distribution of tight junction proteins ZO-1 and occludin [29]. Collectively, our results indicate that ZnONPs possess a unique feature of disrupting both endothelial tight and adherens junctions, similar to titanium dioxide nanoparticles. Importantly, our in vitro findings were demonstrated in vivo, in that repeated pulmonary exposure at various doses for 4 weeks reduced junctional β-catenin and claudin-5 in the arteriolar and alveolar endothelial cell-cell junctions in a dose-dependent manner (Figure 8 and Figure 9), and correlated with enhanced infiltration of leukocytes, pulmonary fibrosis and vascular remodeling (Figure 1 and Figure 2).

VEGF-VEGFR2 signaling regulates endothelial cell proliferation, migration and permeability, and is essential for angiogenesis, vascular homeostasis and various diseases [20,30]. Activation of VEGF-VEGFR2 axis has been shown to facilitate VE-cadherin internalization [20,21]. Intriguingly, we found that ZnONPs-induced permeability and β-catenin dissociation might be independent of VEGF, despite the fact that ZnONPs increased VEGFR2 expression and activation (Figure 6). The molecular pathways that mediate ZnONPs’ induction and activation of VEGFR2 are unclear and require further investigation. 

We have shown that single or repeated exposure of ZnONPs induces pulmonary inflammation, accompanied by an activated AhR pathway in the bronchial and bronchiolar epithelial cells and macrophages in the mouse lungs [6]. Inflammation is one of the early events in inducing endothelial dysfunction. Indeed, ZnONPs increased endothelial ICAM-1 expression that was mediated via an inflammatory AhR pathway (Figure 7), suggesting that ICAM-1 regulation in endothelial cells might also be mediated via a cytokine-IDO1-AhR loop, as we reported in epithelial cells and macrophages [6]. Nevertheless, it appeared that the AhR pathway was not likely to be involved in ZnONPs-mediated alterations of tight and adherens junctions (Figure 7). Our results suggest that ZnONPs alter endothelial cell functions via AhR-dependent and independent mechanisms. An interesting finding was that, unlike tight and adherens junction proteins we have examined, ZnONPs did not affect PECAM-1 level at endothelial cell-cell junctions (Figure 8). Given that PECAM-1 is required for transendothelial migration of leukocytes [23], it is not surprising that PECAM-1 was not affected at the junctions. Collectively, it is tempting to speculate that, in response to the ZnONPs stimulation, ICAM-1 and PECAM-1 facilitate leukocyte adhesion and subsequent transcellular migration. On the other hand, ZnONPs impaired cell-cell junctions, facilitating leukocyte diapedesis through paracellular junctions. Together with our previous findings [6], it is conceivable that ZnONPs have adverse effects not only on pulmonary epithelial cells and macrophages, but also on vascular endothelial cells, leading to lung injury.

## 4. Materials and Methods 

### 4.1. Mouse Model and Histological Analysis

All experimental procedures were performed in accordance with the NIH guidelines (Guide for the care and use of laboratory animals) and approved by the Institutional Animal Care and Use Committee of National Health Research Institutes, Taiwan (#NHRI-IACUC-104016-A, approved on 10 March 2015). Wild-type mice (C57BL/6JNarl) were purchased from the National Laboratory Animal Center, Taiwan and acclimated for 2 weeks at the National Health Research Institutes (Taiwan) animal facility under a 12 h light/dark cycle at 23 ± 1 °C, with a relative humidity of 39–43%. Water and food were provided ad libitum. Mice were subjected to oropharyngeal aspiration of ZnONPs (0, 3, 10 and 30 µg/mouse), twice a week for 4 weeks, and the lungs harvested as we described previously [6]. Animals were killed through isoflurane inhalation, lungs were lavaged with saline, and then carefully dissected and fixed in 10% neutral buffered formalin for 48 h, before processing (including dehydration and clearing) and embedding in paraffin. Serial 5-µm sections were collected. Lung sections were stained with H&E for general histopathological examinations. Masson’s trichrome staining (Sigma, St. Louis, MO, USA) was utilized to evaluate collagen deposition. For immunohistochemistry, sections were treated with heated citrate buffer (ScyTek Laboratories, Logan, UT, USA) for 20 min, before blocking with 10% goat serum. Subsequently, sections were incubated overnight at 4 °C with primary antibodies against CD31 (Cell Signaling Technology, Danvers, MA, USA, rabbit, #77699), claudin-5 (Thermo Scientific, Waltham, USA, mouse, 35-2500), β-catenin (BD Bioscience, mouse, 610154), or CD45 (BD Biosciences, San Jose, CA, USA, rat, 550539). Sections were then incubated with secondary Alexa Flour (Thermo Scientific) or polymer-peroxidase conjugated (DakoCytomation, Glostrup, Denmark) antibodies. For the latter, diaminobenzidine (DakoCytomation) was subsequently used for the visualization of target proteins, followed by hematoxylin for counterstaining (Muto Pure Chemicals, Tokyo, Japan). 

Olympus microscope (BX51, DP71 with objectives 40x/0.9) was used to take histological images. For each mouse, four fields of view (0.4 mm by 0.33 mm) from each left and right lung sections were chosen to quantify claudin-5 expression level in alveoli by color deconvolution using ImageJ software. Eight mice from each condition were used for the quantification of claudin-5 expression. For each field, OD was estimated by the formula OD = log (max intensity/mean intensity) [31]. In statistical analysis, the OD average of these eight fields from each mouse was used. All fields were chosen so that there were no bronchus, large vessel, or arteriole. For the groups treated with or without 3 μg ZnONPs, the fields of view were randomly chosen; for the group treated with 10 or 30 µg ZnONPs, the fields were randomly chosen among the affected areas where alveolar septa were thickened. UPlanSApo 100X/1.45 was used for double immunohistochemistry of CD31 and β-catenin.

To quantify leukocytes infiltration in the lungs, 3–4 cropped fields of views of alveoli (0.19 mm wide square cropped from each 0.4 mm by 0.33 mm view, UPlanSApo 40X/0.9) from each left and right lung were chosen for each mouse. To quantify leukocytes infiltration in the perivascular space of arterioles, 6–10 perivascular spaces (cropped squares in 0.09~0.14 mm width, depending on arteriolar diameters; UPlanSApo 40X/0.9) were chosen for each mouse. Cells were counted manually with image J. Alveolar septal width was manually assessed in Image J following application of a 25-point grid to the field views. Septal width was measured at thirteen standardized cross-points using the line selection tool. For each mouse, 3–4 fields of views (0.4 mm by 0.33 mm, UPlanSApo 40X/0.9) from each left and right lung were used. Arteriolar wall thickness was manually measured in Image J. Arterioles selected for measurement were 17–85 μm in diameter. For each mouse, an average value was obtained from 6~16 arterioles (UPlanSApo 40X/0.9).

### 4.2. ZnONPs and Cell Culture

ZnONPs were purchased from NanoScale Corporation (Manhattan, KS, USA) and prepared as irregular pillars with sizes of 9.1 ± 1.9 nm, as described previously [32]. HUVECs (Lonza, Basel, Switzerland) were maintained in endothelial cell growth medium-2 (EGM-2) (Lonza) and passages 5–6 were used for experiments. In some experiments, to exclude the effects of VEGF, HUVECs were initially cultured in EGM-2 to the designated confluence and then starved in starvation medium, which is EGM-2 without certain growth factors (hFGF-B, R3-IGF-1, hEGF and VEGF) and with only 0.5%, instead of the normal 2% FBS for 17 h, prior to treatment with ZnONPs. In the experiments with DMF (Sigma), cells were pretreated with or without 10 μM DMF for 30 min, prior to ZnONPs treatment.

### 4.3. Cell Viability Assay

HUVECs were seeded onto 96-well plates at a density of 1.2 × 10^5^ cells/well and cultured for 24 h before treatment with ZnONPs (0, 5, 10, 15, 20, 25, 30 and 50 µg/mL) in triplicate. After 24 h incubation, cells were refreshed with 1 mg/mL 3-(4,5-dimethyl-2-thiazolyl)-2,5-diphenyl-2H-tetrazolium bromide (MTT, Sigma) in EGM-2 and incubated for an additional 2 h before viability assessed. The crystals were dissolved in DMSO (200 μL/well) and optical density (OD) measured at 570 nm. Untreated control was set as 100%. Four independent experiments were performed.

### 4.4. Permeability Assay

HUVECs were seeded onto Corning^®^ Transwell^®^ polycarbonate membrane cell culture inserts (0.4 μm pore size) at the density of 1.1 × 10^5^ cells/insert in triplicate and cultured in endothelial cell growth basal medium-2 (EBM-2) for 72 h to reach confluence. Cells were then treated with indicated non-cytotoxic concentrations of ZnONPs or IL-1β (as a positive control, 10 ng/mL, ProSpec-Tany TechnoGene). After 24 h, the upper chamber was refreshed with a 500 μL medium containing 1 mg/mL FITC-Dextran (Sigma, FD70S), and a lower chamber with 1.5 mL medium, so that only diffusive force was involved in solute permeability. After 30 min incubation, the fluorescence of the lower chamber was measured with Infinite M200 PRO microplate reader (TECAN). Untreated control was set as 1. Three independent experiments were performed.

### 4.5. Western Blot Analysis

HUVECs were seeded onto 35 mm dishes (1.5 × 10^6^ cells/dish) and incubated for 48 h to confluence, before cells treated with 0, 10, 15, 20 μg/mL ZnONPs in 2 mL of medium per dish. After 24 h treatment, cells were washed quickly with cold PBS and lysis buffer (25 mM Tris pH 7.4, 150 mM NaCl, 0.5% Na deoxycholate, 2% NP-40, 0.2% SDS, 7% glycerol) containing protease and phosphatase inhibitors was added to the dish. Cells were scraped into a 1.5-mL tube and incubated on ice for 30 min, before centrifugation to collect supernatant as total protein extract. Protein extracts were aliquoted in small volume and stored in −80 °C. Total proteins were subjected to SDS polyacrylamide gel electrophoresis and proteins transferred to PVDF membrane using standard wet transfer method (22 volt, overnight in a cold-room). The blots were probed with primary antibodies against ICAM-1 (Santa Cruz, Dallas, TX, USA, mouse, sc8439; R&D Systems, Minneapolis, MA, USA, goat, AF796), VCAM-1 (Santa Cruz, rabbit, sc8304), VE-cadherin (abcam, rabbit, ab33168), ZO-1 (Cell Signaling, mouse, #8193), claudin-5 (Thermo Scientific, mouse, 35-2500), VEGFR2 (Cell Signaling, rabbit, #2479), or phospho-VEGFR2 (p-VEGFR2; Tyr1175; Cell Signaling, rabbit, #3770). For loading control, blots were subsequently probed with actin (Millipore, Burlington, VT, USA, mouse, MAB1501) or GAPDH (GeneTex, Irvine, CA, USA, rabbit, GTX100118). The protein bands were quantified by Image J and normalized to control without ZnONPs.

### 4.6. Immunocytochemistry

HUVECs (1.71 × 10^5^ cells/cm^2^) were seeded onto gelatin-coated glass coverslips and cultured for 48 h to confluence, followed by treatment of ZnONPs for 24 h. Cells were then fixed in cold 4% paraformaldehyde for 15 min, permeabilized in 0.5% tween-20 for 30 min at room temperature, blocked with 10% goat serum for 1 h, and then incubated overnight at 4 °C with primary antibodies VE-cadherin (BD Biosciences, mouse, Clone 75 for EC1 domain, 610252 or abcam, rabbit, ab33168 for C-terminal domain) and β-catenin (BD Biosciences, mouse, 610154) for double immunostaining. For claudin-5 (abcam, rabbit, ab15106) and ZO-1 (Thermo Scientific, mouse, 33-9100), fixation and permeabilization were performed with −20 °C methanol and acetone in 1:1 ratio on ice. Alexa Fluor secondary antibodies (Thermo Scientific) and DAPI (Sigma) were used to visualize the proteins of interest and nuclei, respectively. All images were taken with a fluorescence microscope and a high-resolution digital camera (Olympus BX51, DP71). UPlanSApo 60X/1.35 and UPlanSApo 100X/1.45 were used for tight junction and adherens junction proteins respectively. Light intensity was adjusted so that exposure times were 1/1.3–1/5 seconds in all experiments. Within one experiment, exposure time was fixed across all conditions. All immunocytochemistry data had been repeated in at least three independent experiments.

### 4.7. Statistical Analysis

Data are presented as mean ± SE of at least three independent experiments and analyzed statistically by Student’s t-test. *p* values < 0.05 are considered statistically significant.

## 5. Conclusions

**Figure 10:**
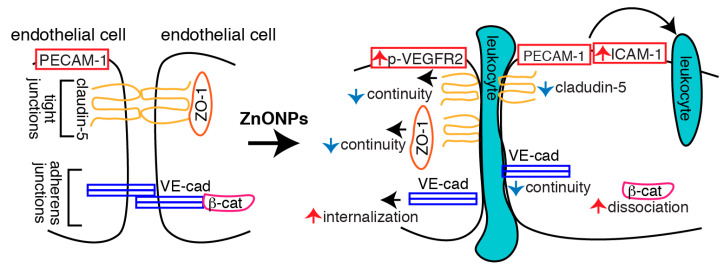
Schematic illustration of the actions of ZnONPs on endothelial cells. ZnONPs increase ICAM-1 expression, together with PECAM-1 might facilitate leukocyte adhesion and transmigration into the vessel wall. ZnONPs not only decrease claudin-5 expression but also cause discontinuity of claudin-5 and ZO-1 at the junctions; these collectively disrupt endothelial cell-cell tight junctions. ZnONPs activate VEGFR2 and elicit VE-cadherin internalization, accompanied with discontinuous distribution of VE-cadherin at the junctions and dissociation of β-catenin from VE-cadherin, resulting in disassembly and destabilization of adherens junctions. Disruptions of both tight and adherens junctions lead to increased endothelial barrier dysfunction and permeability, which might facilitate leukocyte diapedesis via paracellular junctional opening.

In conclusion, we demonstrated that ZnONPs induce endothelial dysfunction by increasing inflammatory adhesion molecule ICAM-1 expression and disrupting both tight and adherens junctions (Figure 10). Increased ICAM-1 level might facilitate leukocyte adhesion and transmigration into the vessel wall. Decreased claudin-5 expression, together with discontinuous junctional distribution of claudin-5 and ZO-1, impairs endothelial tight junctions following ZnONPs exposure. Increased VE-cadherin internalization, accompanied with discontinuity of VE-cadherin and dissociation of β-catenin, results in disassembly and destabilization of adherens junctions. ZnONPs-elicited alterations of tight and adherens junction proteins compromise the integrity and stability of the junction network, leading to endothelial barrier dysfunction and increased permeability, which might in turn facilitate leukocyte diapedesis via paracellular junctional opening (Figure 10). Our results highlight the susceptibility of vascular endothelial barrier to ZnONPs exposure and emphasize the potential risk of ZnONPs on vascular functions. Although ZnONPs have been extensively developed for use in the consumer and biomedical fields [1], ZnONPs may be a double-edged sword. Therefore, exposure to ZnONPs in many different settings should be carefully evaluated for their vascular effects and subsequent health impacts.

## Supplementary Materials

The following are available online at https://www.mdpi.com/1422-0067/21/10/3437/s1.

## Abbreviations

AhR	Aryl hydrocarbon receptor
DMF	3′,4′-dimethoxyflavone
EGM2	endothelial cell growth medium-2
HUVECs	human umbilical vein endothelial cells
ICAM-1	intercellular adhesion molecule-1
IDO1	indoleamine 2,3-dioxygenase
MTT	3-(4,5-dimethyl-2-thiazolyl)-2,5-diphenyl-2H-tetrazolium bromide
VCAM-1	vascular cell adhesion molecule-1
VE-cadherin	vascular endothelial cadherin
VEGF	vascular endothelial growth factor
VEGFR2	vascular endothelial growth factor receptor 2
ZnONPs	zinc oxide nanoparticles
ZO-1	zonula occludens-1

## References

[1] Jiang J., Pi J., Cai J. (2018). The advancing of zinc oxide nanoparticles for biomedical applications. Bioinorg. Chem. Appl..

[2] Hou J., Wu Y., Li X., Wei B., Li S., Wang X. (2018). Toxic effects of different types of zinc oxide nanoparticles on algae, plants, invertebrates, vertebrates and microorganisms. Chemosphere.

[3] Monse C., Raulf M., Hagemeyer O., Van Kampen V., Kendzia B., Gering V., Marek E.M., Jettkant B., Bunger J., Merget R. (2019). Airway inflammation after inhalation of nano-sized zinc oxide particles in human volunteers. BMC Pulm. Med..

[4] Liu H., Yang H., Fang Y., Li K., Tian L., Liu X., Zhang W., Tan Y., Lai W., Bian L. (2020). Neurotoxicity and biomarkers of zinc oxide nanoparticles in main functional brain regions and dopaminergic neurons. Sci. Total Environ..

[5] Singh N., Das M.K., Gautam R., Ramteke A., Rajamani P. (2019). Assessment of intermittent exposure of zinc oxide nanoparticle (ZNP)-mediated toxicity and biochemical alterations in the splenocytes of male Wistar rat. Environ. Sci. Pollut. Res. Int..

[6] Ho C.C., Lee H.L., Chen C.Y., Luo Y.H., Tsai M.H., Tsai H.T., Lin P. (2017). Involvement of the cytokine-IDO1-AhR loop in zinc oxide nanoparticle-induced acute pulmonary inflammation. Nanotoxicology.

[7] Vermette D., Hu P., Canarie M.F., Funaro M., Glover J., Pierce R.W. (2018). Tight junction structure, function, and assessment in the critically ill: A systematic review. Intensive Care Med. Exp..

[8] Dejana E. (2004). Endothelial cell-cell junctions: Happy together. Nat. Rev. Mol. Cell Biol..

[9] Ohta H., Chiba S., Ebina M., Furuse M., Nukiwa T. (2012). Altered expression of tight junction molecules in alveolar septa in lung injury and fibrosis. Am. J. Physiol. Lung Cell Mol. Physiol..

[10] Greene C., Hanley N., Campbell M. (2019). Claudin-5: Gatekeeper of neurological function. Fluids Barriers CNS.

[11] Van Itallie C.M., Fanning A.S., Bridges A., Anderson J.M. (2009). ZO-1 stabilizes the tight junction solute barrier through coupling to the perijunctional cytoskeleton. Mol. Biol. Cell.

[12] Vestweber D. (2002). Regulation of endothelial cell contacts during leukocyte extravasation. Curr. Opin. Cell Biol..

[13] Gotsch U., Borges E., Bosse R., Boggemeyer E., Simon M., Mossmann H., Vestweber D. (1997). VE-cadherin antibody accelerates neutrophil recruitment in vivo. J. Cell Sci..

[14] Corada M., Liao F., Lindgren M., Lampugnani M.G., Breviario F., Frank R., Muller W.A., Hicklin D.J., Bohlen P., Dejana E. (2001). Monoclonal antibodies directed to different regions of vascular endothelial cadherin extracellular domain affect adhesion and clustering of the protein and modulate endothelial permeability. Blood.

[15] Sukriti S., Tauseef M., Yazbeck P., Mehta D. (2014). Mechanisms regulating endothelial permeability. Pulm. Circ..

[16] Blaise S., Polena H., Vilgrain I. (2015). Soluble vascular endothelial-cadherin and auto-antibodies to human vascular endothelial-cadherin in human diseases: Two new biomarkers of endothelial dysfunction. Vasc. Med..

[17] Claesson-Welsh L. (2015). Vascular permeability—The essentials. Upsala J. Med. Sci..

[18] Sun J., Wang S., Zhao D., Hun F.H., Weng L., Liu H. (2011). Cytotoxicity, permeability, and inflammation of metal oxide nanoparticles in human cardiac microvascular endothelial cells: Cytotoxicity, permeability, and inflammation of metal oxide nanoparticles. Cell Biol. Toxicol..

[19] Blankenberg S., Barbaux S., Tiret L. (2003). Adhesion molecules and atherosclerosis. Atherosclerosis.

[20] Weis S.M., Cheresh D.A. (2005). Pathophysiological consequences of VEGF-induced vascular permeability. Nature.

[21] Gavard J., Gutkind J.S. (2006). VEGF controls endothelial-cell permeability by promoting the β-arrestin-dependent endocytosis of VE-cadherin. Nat. Cell Biol..

[22] Feng D., Nagy J.A., Pyne K., Dvorak H.F., Dvorak A.M. (2004). Ultrastructural localization of platelet endothelial cell adhesion molecule (PECAM-1, CD31) in vascular endothelium. J. Histochem. Cytochem..

[23] Muller W.A., Weigl S.A., Deng X., Phillips D.M. (1993). PECAM-1 is required for transendothelial migration of leukocytes. J. Exp. Med..

[24] Chen I.C., Hsiao I.L., Lin H.C., Wu C.H., Chuang C.Y., Huang Y.J. (2016). Influence of silver and titanium dioxide nanoparticles on in vitro blood-brain barrier permeability. Environ. Toxicol. Pharmacol..

[25] Cornu R., Chretien C., Pellequer Y., Martin H., Beduneau A. (2020). Small silica nanoparticles transiently modulate the intestinal permeability by actin cytoskeleton disruption in both Caco-2 and Caco-2/HT29-MTX models. Arch. Toxicol..

[26] Kowalczyk A.P., Nanes B.A. (2012). Adherens junction turnover: Regulating adhesion through cadherin endocytosis, degradation, and recycling. Subcell. Biochem..

[27] Adam A.P. (2015). Regulation of endothelial adherens junctions by tyrosine phosphorylation. Mediat. Inflamm..

[28] Setyawati M.I., Tay C.Y., Chia S.L., Goh S.L., Fang W., Neo M.J., Chong H.C., Tan S.M., Loo S.C., Ng K.W. (2013). Titanium dioxide nanomaterials cause endothelial cell leakiness by disrupting the homophilic interaction of VE-cadherin. Nat. Commun..

[29] Davidson M.K., Russ P.K., Glick G.G., Hoffman L.H., Chang M.S., Haselton F.R. (2000). Reduced expression of the adherens junction protein cadherin-5 in a diabetic retina. Am. J. Ophthalmol..

[30] Carmeliet P., Collen D. (2000). Molecular basis of angiogenesis. Role of VEGF and VE-cadherin. Ann. N. Y. Acad. Sci..

[31] Ruifrok A.C., Johnston D.A. (2001). Quantification of histochemical staining by color deconvolution. Anal. Quant. Cytol. Histol..

[32] Chen J.K., Ho C.C., Chang H., Lin J.F., Yang C.S., Tsai M.H., Tsai H.T., Lin P. (2015). Particulate nature of inhaled zinc oxide nanoparticles determines systemic effects and mechanisms of pulmonary inflammation in mice. Nanotoxicology.

