# Supplementary material for: Exposure to Zinc Oxide Nanoparticles Disrupts Endothelial Tight and Adherens Junctions and Induces Pulmonary Inflammatory Cell Infiltration
Source: Int J Mol Sci. 2020 May 13;21(10):3437. doi: 10.3390/ijms21103437 (PMC7279309; doi:10.3390/ijms21103437)
Supplement: Supplementary file 1 [file ijms-21-03437-s001.pdf]

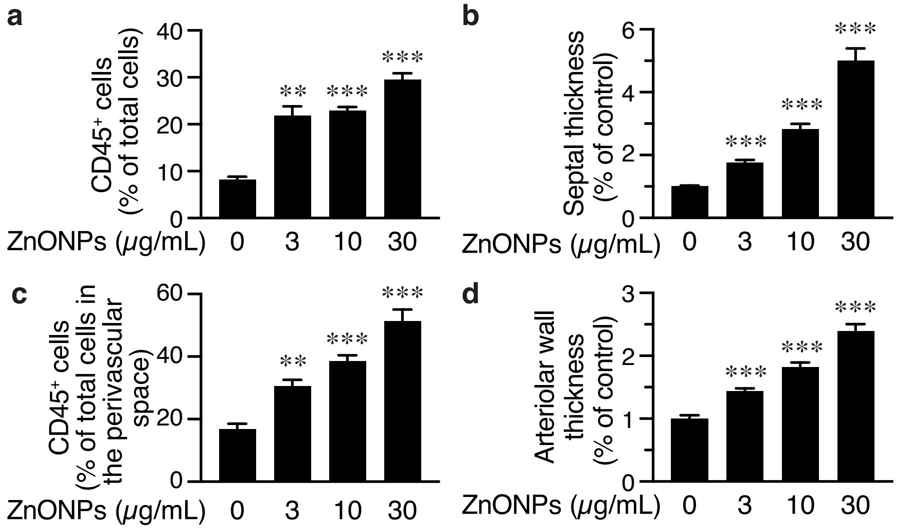


Figure S1. Quantification of inflammatory cell infiltration and morphological changes of alveoli and pulmonary arterioles in mice following repeated exposure to ZnONPs. (**a**) Number of infiltrated leukocytes normalized by the total cell number per field of view. An average from 5-6 fields of view was used for each mouse (n=4 each condition). (**b**) Alveolar septal thickness in mice was quantified, with 6-8 fields of views for each mouse (n=6 each condition). (**c**) Number of infiltrated leukocytes normalized by the total cell number in the perivascular space. An average from 6-10 perivascular spaces was used for each mouse (n=4 each condition). (**d**) Arteriolar wall thickness was quantified (n=5, each). Data are mean ± SE, Student's *t*-test, $**p < 0.01$, $***p < 0.001$ vs. control.
